# Online Backwash Optimization of Membrane Filtration for Produced Water Treatment

**DOI:** 10.3390/membranes9060068

**Published:** 2019-06-05

**Authors:** Kasper L. Jepsen, Mads V. Bram, Leif Hansen, Zhenyu Yang, Steven M. Ø. Lauridsen

**Affiliations:** 1 Department of Energy Technology, Aalborg University Esbjerg, Niels Bohrs Vej 8, DK-6700 Esbjerg, Denmark; klj@et.aau.dk (K.L.J.); mvb@et.aau.dk (M.V.B.); lha@et.aau.dk (L.H.); 2 Total, Britanniavej 10, DK-6700 Esbjerg, Denmark; steven.lauridsen@total.com

**Keywords:** backwash, optimization, produced water treatment, membrane filtration, online

## Abstract

In the offshore oil and gas sector, produced water is discharged into the sea, but increasing environmental concerns and stricter governmental regulations require new technologies to be considered. Membrane filtration is a promising technology to improve separation, but fouling of the membranes causes a significant reduction in flow capacity. To reduce fouling, optimization of the backwashing parameters is given much attention. Comprehensive and time-consuming experiments are used to model the effect of backwashing, but most methods neglect time varying features present in the offshore produced water treatment train. In this paper, a backwashing scheduling algorithm is proposed, which dynamically selects the filtration and backwashing durations to maximize the average net permeate production. The proposed algorithm is tested on a lab-scaled pilot plant, where it was able to adapt as irreversible fouling accumulated and the OiW concentration changed. The paper concludes that the removal rate of oil fouling was observed to be dependent on the rate at which the backwashing pressure could be established. As the proposed method online adapts to the current conditions, it can improve the filtration capacity compared to cases with constant backwashing and filtration durations throughout the lifetime of the facilities.

## 1. Introduction

In the petroleum industry, produced water (PW) is the largest waste stream, especially as the water cut increases with oil field maturity [1,2]. Typically, the PW is treated to comply with Danish regulations, where the oil-in-water (OiW) concentration must be below 30 mg/L before discharged and the total amount of discharged dispersed oil must remain below 222 tonnes annually, regardless of concentration. In 2015, an oil producer discharged 95% of their annual allowed limit, which incentify the need for new methods and technologies to comply with future regulations [3]. Crossflow (CF) membrane filtration is an attractive candidate for improving OiW separation for produced water treatment (PWT). Membrane technology has been investigated for OiW separation, but challenges such as the complexity of the PW, fouling, and required installation footprint complicates industrial implementation. The complexity of PW is especially problematic as the composition varies with respect to well, maturity, and field [4].

Backwashing of the membranes are often deployed to remove accumulated fouling. During backwashing, the transmembrane pressure (TMP) is reversed and produced permeate is used to wash away the accumulated fouling. Typically, fouling is categorized into four groups depending on how the fouling blocks the pores of the membrane. The four groups are commonly defined as: complete blockage, intermediate blockage, standard blockage, and cake blockage [5]. A fouling analysis of an OiW mixture in [6] indicated that the main contributor to fouling was cake blockage. Studies claim that backwashing is effective for fouling removal, thus maintaining an overall higher flux [7,8]. The backwashing method has multiple adjustable parameters, such as duration, interval (time between backwashes), and intensity (reversed flow rate or TMP), affecting the removal efficiency. In most cases, the parameters are either ad hoc or based on a pre-investigation of a limited set of different durations and intervals [8,9,10,11]. Unnecessary backwashing wastes both permeate and filtration time, reducing the overall capacity of the filtration system.

A method to increase filtration capacity is to schedule backwashing based on the TMP (TMP-based) or flux (flux-based), rather than a fixed interval (time-based). The TMP-based and flux-based methods allow for some adaptation, where the incremental accumulation of irreversible fouling would cause the backwashing frequency to gradually increase. Results in [12] show that the TMP-based method can maintain the same flux as the time-based method, but with a 25% reduction in backwashing media due to the reduction in backwashing duration and interval.

More advanced methods were studied in [13,14,15], where artificial neural network (ANN), response surface methodology (RSM), and run-to-run control were applied, respectively. The ANN was trained to predict system performance with variations in duration and interval. Based on the ANN, the optimal duration for the filtration and backwashing phase could be determined in [13]. In [14], the RSM was deployed to model the net production over a range of different parameters such as backwashing intervals and durations. Both the RSM and ANN studies select a fixed duration for both the filtration phase and the backwashing phase to be used throughout the lifetime of the system, not allowing adaptation without re-identification of the model. However, the run-to-run control methodology adapts to time varying conditions by utilizing measurements from previous filtration cycles to adjust the backwashing parameters, consequently reducing energy usage for membrane filtration by up to 20% according to simulations [15].

In this paper, a method for maximizing net permeate production with respect to backwashing and filtration durations will be proposed. The method will adapt to process changes, such as feed concentration and irreversible fouling by online estimating of the optimal backwashing and filtration durations with respect to net permeate production. The suggested algorithm is data-driven, thus avoiding model estimation. The proposed algorithm is implemented and validated on a pilot plant, and the results show the algorithm adapting as irreversible fouling accumulates.

## 2. Experimental

This section is divided into two parts: Firstly, the lab-scale pilot plant used for experiments and validation is described. Secondly, the experimental conditions, OiW mixture, and operation are described.

### 2.1. Pilot Plant

The pilot plant is developed to emulate process control related problems for offshore topside OiW separation [16]. The plant consists of a supply system, horizontal pipeline, vertical riser, gravity-based separators, hydrocyclones, and CF membrane filtration, but only the supply system and membranes are used in this study. The filtration system consists of 24 membranes, each with a membrane surface area of 0.34 m${}^{2}$, divided into eight filtration units each capable of parallel or serial configuration. The deployed membranes are made of silicon carbide (SiC) with a nominal pore size of 0.04 μm (ultrafiltration) and are produced by LiqTech. The SiC membranes are well known for their chemical stability, narrow pore size distribution, high flux, and hydrophilic properties. The hydrophilic properties of the membrane ensure that oily particles and oil droplets are not absorbed by the membranes [17]. Furthermore, based on the selected pore size, the pilot plant should be able to reduce the OiW concentration to less than 3 mg/L [18].

**Figure 1:**
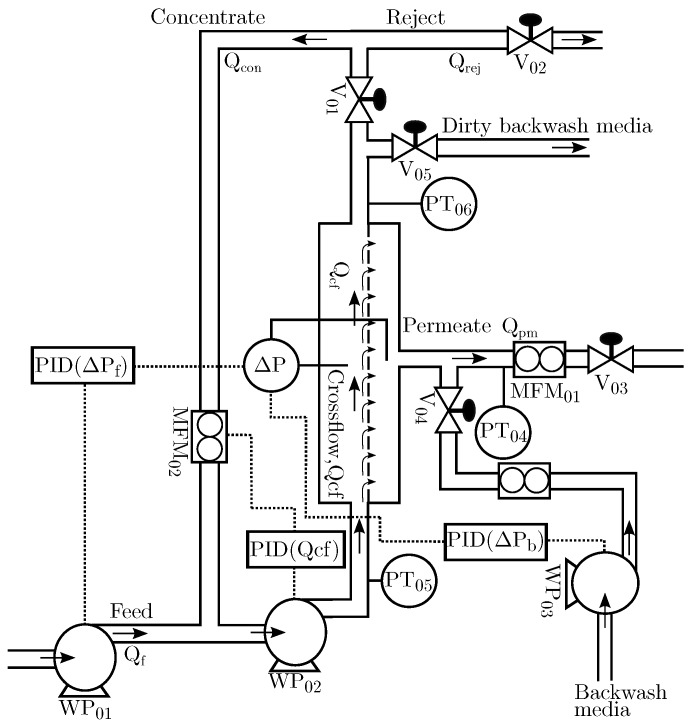
Piping and instrumentation diagram, where the syntax PID($Q_{cf}$) means that $Q_{cf}$ is the controlled variable, where $\Delta P_{f}$ and $\Delta P_{b}$ are the TMP references during filtration and backwashing.

Figure 1 shows an overview of the membrane filtration unit used in this study. The CF pump (${WP}_{02}$) is delivering crossflow velocities (CFV) up to 2.553 m/s, and the feed pump (${WP}_{01}$) is used to control the TMP.

The pilot plant is equipped with both Jorin Visual Process Analyser (ViPA) (Leicestershire, UK) and Turner Designs TD-4100XD (Fresno, CA, USA). The ViPA analyses droplet size and shape, and provides a concentration estimate, based on video microscopy. The Turner Designs TD-4100XD is a fluorescence-based monitor that measures the OiW concentration online. The two analyzers have been used and results seem promising, but the instruments should be validated using industrial approved standard methods, such as the gas chromatography with flame ionization detector (GC-FID) [19,20].

Matlab Simulink Real-Time (R2016b, MathWorks, Natick, MA, USA) is selected as the implementation platform for fast and flexible prototyping. Matlab is connected to the sensors and actuators of the pilot plant through National Instruments (Austin, TX, USA) and Speedgoat (Bern, Switzerland) I/O-cards. The entire system is sampled at 100 Hz, providing the opportunity to study relatively fast process dynamics.

### 2.2. Experimental Conditions

**Figure 2:**
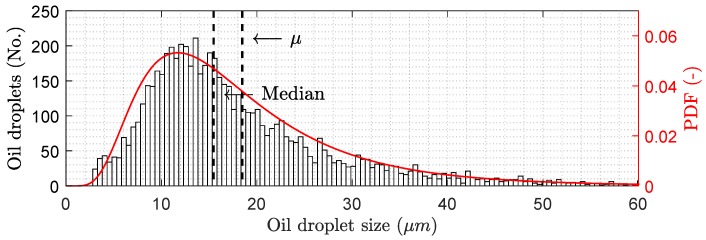
Droplet size distribution histogram based on measurements from the ViPA monitor with a mean and median of 18.47μm and 15.47μm, respectively [21].

As the PW quantity demanded by the experiment poses a safety and fire risk, the feed mixture used in this study is not equivalent to offshore PW, but rather a refined oil mixed with tap water. Based on pilot plant experience, a normal grade motor oil contains surfactants to a degree where the mixture becomes milky white and the mixture becomes difficult to separate for disposal. As such, the feed mixture consists of tap water and non-detergent SAE 30 motor oil from Midland (Riga, Latvia). The OiW mixture has a natural tendency to separate in the feed tank, therefore two large mechanical stirrers from Milton Roy mixing (Houston, TX, USA) are deployed to ensure proper and consistent oil and droplet size distribution in the feed tank (2 m${}^{3}$ mixture). The speed of the stirrers can be controlled to either decrease or increase the droplet size. The mechanical stirrers have a combined electrical effect of 2.2 kW and a mixing intensity of 1469 m${}^{3}$/h. The droplet size distribution during stirring was analyzed in [21] and is shown in Figure 2. Based on the results, 95% of the droplets are between 5.4 μm and 46.6 μm, which are close to the 5 μm–50 μm range reported in [22]. Real PW contains a varying degree of natural surfactants depending on well location, posing a challenge to the separation train. In particular, the difference between the feed mixture and real PW is a known limitation of the pilot plant. Ideally, real PW should have been used, but the quantity required poses a safety risk which must be dealt with before real PW can be introduced into the pilot plant.

To ensure relatively constant concentration throughout the experiments, the permeate, reject, and backwashing media are returned to the feed tank except for a small amount of permeate that is kept in a separate tank to be used for backwashing. The ratio between the total feed volume and the permeate stored for backwashing is over 10, thus reducing the oscillation in OiW concentration that occurs as a consequence. The feed concentration is intentionally higher than the expected concentration in PW to artificially increase the fouling growth. According to the TD-4100XD, the mean concentration of feed is 1277 mg/L.

The active controllers and the control pairings used throughout this study are illustrated in Figure 1.

## 3. Results and Discussion

In this section, the scheduling strategy is described and modified based on the observed fouling behavior. The method is discretized and implemented on the pilot plant, and lastly the results are presented and discussed.

### 3.1. Scheduling Strategy

**Figure 3:**
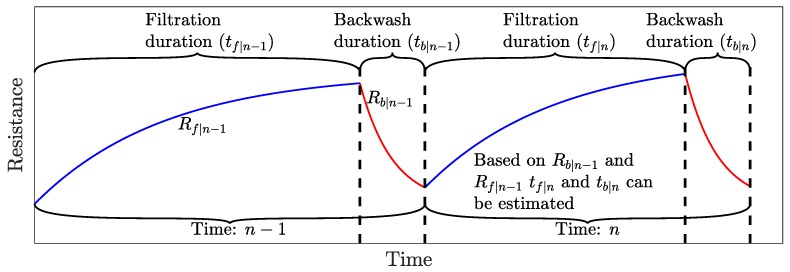
Resistance trend and notations.

Fouling occurs during filtration and increases the permeate flow resistance, but commonly fouling is partly removed by backwashing, as illustrated in Figure 3. The proposed example is to model how the resistance develops over a filtration cycle (*n*), and, based on the previously estimated resistance models, $R_{f|n-1}$ and $R_{b|n-1}$, the current filtration and backwashing duration can be selected such that the net permeate production is maximized.

The considered optimization problem is defined as the maximization of net permeate production over a filtration cycle, which is formulated as:

(1) $$\max_{t_{f|n},t_{b|n}\in [0,\infty [}j_{avg|n}\left(t_{f|n},t_{b|n}\right),$$

where $t_{b|n}$ and $t_{f|n}$ are the backwashing and filtration durations for the current filtration cycle, respectively, and $j_{avg|n}$ is the average flux over the filtration cycle:

(2) $$j_{avg|n}=\frac{\int_{0}^{t_{f|n}}j_{f|n}\left(t\right)\;dt-\int_{t_{f|n}}^{t_{f|n}+t_{b|n}}j_{b|n}\left(t\right)\;dt}{t_{f|n}+t_{b|n}},$$

where $j_{f|n}\left(t\right)$ and $j_{b|n}\left(t\right)$ are the flux during filtration and backwashing for cycle no. *n*. Assuming steady state, Darcy’s law can be used to describe the relationship between flux, TMP, and resistance [9,23]:

(3) $$j=\frac{\Delta P}{R},$$

where *j* is the permeate flux, ΔP is the TMP, and *R* is the permeate resistance for 1 $m^{2}$ membrane area. Darcy’s law can estimate the resistance during periods where the changes in resistance is significantly slower than the hydro-, valve, and pump dynamics. However, the estimated resistance is inaccurate during transient periods. As the resistance model for the current cycle is unknown, the resistance models estimated based on the previous cycle, $R_{b|n-1}\left(t\right)$ and $R_{f|n-1}\left(t\right)$, can be used to predict fouling behavior for the current cycle. Assuming constant pressure filtration and applying Darcy’s law, Equation (2) can be reformulated as:

(4) $$\begin{array}{cc} j_{avg|n}= & \frac{\Delta P_{b}\int_{0}^{t_{f|n}}\frac{1}{R_{f|n-1}\left(t\right)}\;dt}{t_{f|n}+t_{b|n}}+\frac{\Delta P_{f}\int_{t_{f|n}}^{t_{f|n}+t_{b|n}}\frac{1}{R_{b|n-1}\left(t\right)}\;dt}{t_{f|n}+t_{b|n}}, \end{array}$$

where $\Delta P_{f}$ and $\Delta P_{b}$ are the pressures applied during the filtration and backwashing phases, respectively.

### 3.2. Backwash Resistance Model

In order to maximize Equation (1), the resistance models $R_{f}$ and $R_{b}$ must be identified, where it is anticipated that $R_{f}$ and $R_{b}$ are linear and exponential functions of time, respectively [15,24].

**Figure 4:**
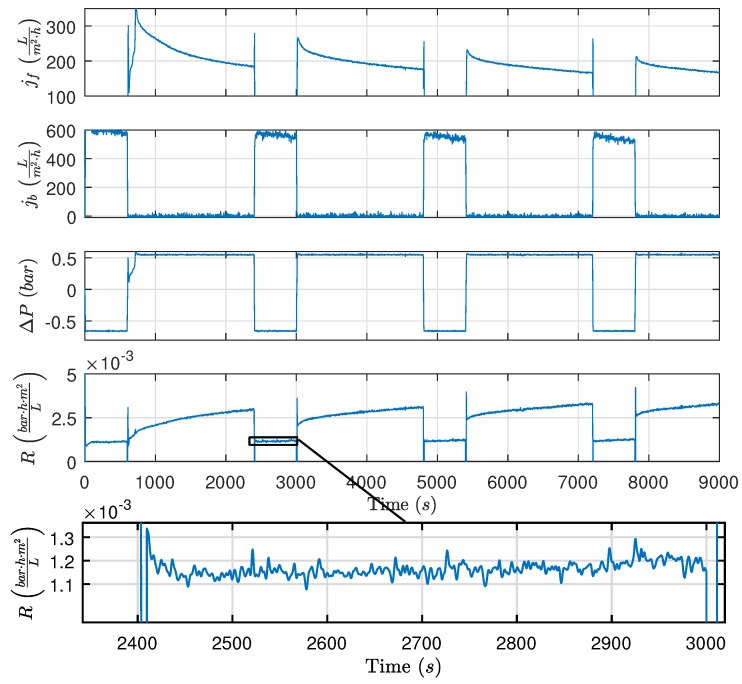
Highlighting resistance for both the filtration and backwashing phase. Experimental conditions: $t_{f}=1800\;s,t_{b}=600\;s,Q_{cf,ref}=1\;\frac{m}{s}$, $\Delta P_{f,ref}=0.5\;\mathrm{bar}$, and $\Delta P_{b,ref}=-0.6\;\mathrm{bar}$, where the subscript ref denotes the control reference for the given variable, and $Q_{cf}$ is the CFV.

Figure 4 shows the estimated resistance, flux, and pressure during filtration and backwashing operation. The fouling behavior during the filtration phases behaved as expected based on different studies addressing fouling of membranes treating oily waste water [10,25,26,27,28,29]. However, the resistance during backwashing did not behave as theorized, indicating that backwashing has no apparent effect on the estimated resistance. Based on the experiment, the estimated resistance is non-decreasing during backwashing, whereas the subsequent filtration phase shows that the permeate resistance has been reduced during the backwashing phase. Since the reduction in resistance caused by backwashing only is observed once filtration is reestablished, the dynamic behavior is difficult to identify. It is conceivable that the removable fouling is removed during the transient period between filtration and backwashing; consequently, the dynamics from the valves and pumps obscure the dynamic behavior of the permeate flow resistance. The same fouling behavior was observed in [30] where municipal wastewater was treated, indicating that the observed fouling behavior does indeed exist outside the laboratory setup. Furthermore, the backwashing durations in several different studies indicate that it is possible that oil fouling of the ceramic membranes is removed during the transient period between filtration and backwashing [7,8,9,31]. However, none of the studies explicitly study the flux recovery as a function of backwashing duration.

**Figure 5:**
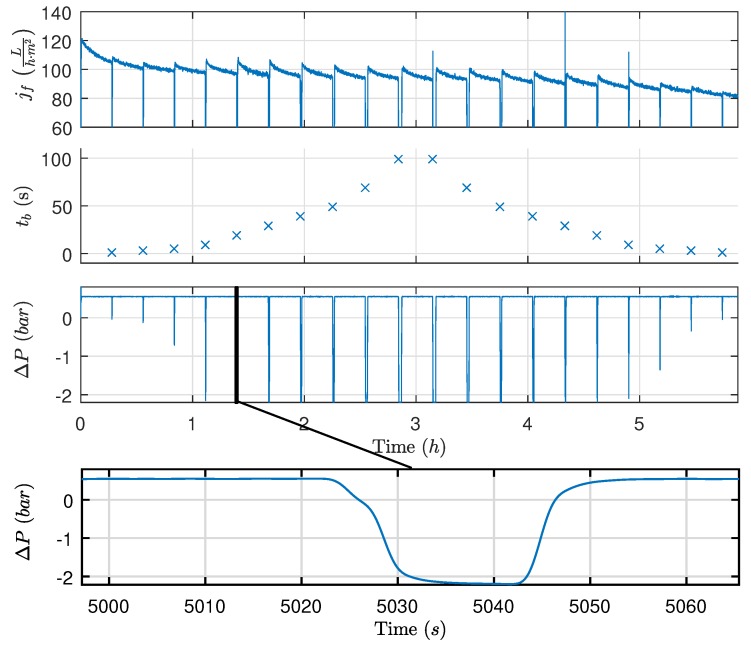
Experiment with different backwashing durations to identifying backwashing behavior. Experimental conditions: $t_{f}=1000\;s,Q_{cf,ref}=1\;\frac{m}{s}$, $\Delta P_{f,ref}=0.55\;\mathrm{bar}$, and $\Delta P_{b,ref}=-2.2\;\mathrm{bar}$.

**Figure 6:**
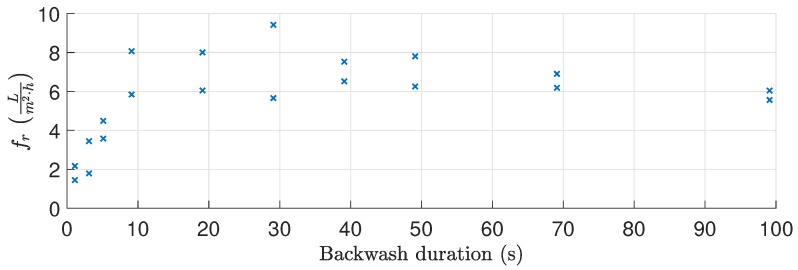
Flux recovered as a function of backwashing duration as defined in Equation (5), based on the data illustrated in Figure 5.

Since the reduction in resistance caused by backwashing can be observed once filtration is reinitiated, a set of different backwashing durations (1 s to 100 s) were tested to investigate the relationship between backwashing duration and recovered flux, where the recovered flux is defined as:

(5) $$f_{r}=\frac{\int_{t_{b|n}}^{t_{b|n}+\Delta t}j_{f|n}\left(t\right)dt}{\Delta t}-\frac{\int_{t_{f|n-1}-\Delta t}^{t_{f|n-1}}j_{f|n-1}\left(t\right)dt}{\Delta t},$$

where Δt is 10 s to reduce the impact of noise on the estimated recovered flux. Figure 5 shows the backwashing experiment, whereas the recovered flux for each backwashing duration is shown in Figure 6. For the backwashing durations (>10 s), the backwashing pressure controller is unable to establish the reference pressure before termination.

**Figure 7:**
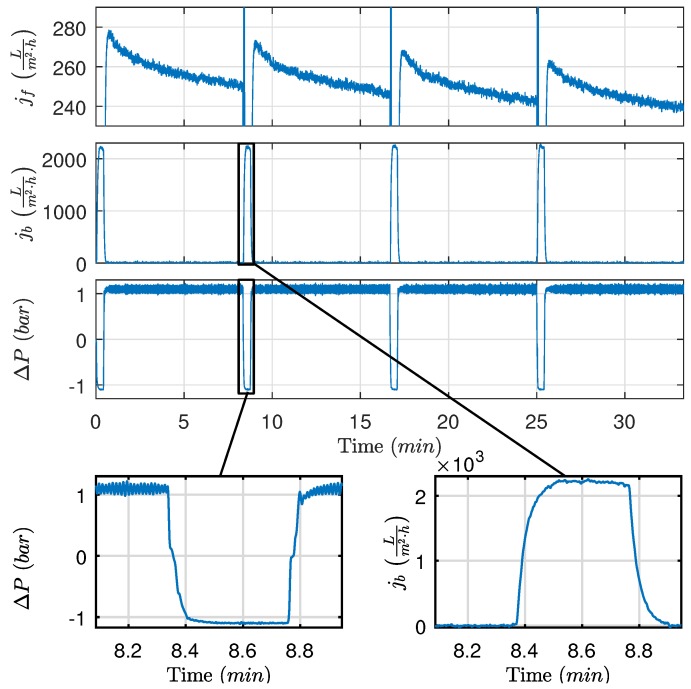
Experiment showing much higher backwashing flow compared to permeate flow at identical pressure. Experimental conditions: $t_{f}=475\;s,t_{b}=25\;s,Q_{cf,ref}=1\;\frac{m}{s}$, $\Delta P_{f,ref}=1.1\;\mathrm{bar}$, and $\Delta P_{b,ref}=-1.1\;\mathrm{bar}$.

Based on results, the recovered flux remains relatively constant for backwashing durations between 10 s and 100 s. Approximately 10 s is required to achieve the desired pressure and the highest degree of recovery in flux, implying that the removable oil fouling is quickly removed once the desired pressure is achieved or removed during the pressure building phase. Furthermore, as shown in Figure 7, the backwashing flux can be eight times higher than the filtration flux when identical drive pressures are applied. As the large difference in flow rate is only present once fouling has accumulated, it is theorized that the fouling behaves similar to a check valve, where fouling is temporarily pushed away only to move back once the flow direction is reversed. It is conceivable that, by increasing the shear rate and thereby backwashing pressure, more fouling could be dislodged from the membranes. However, the pilot plant is unable to deliver higher pressure and extensive modification is required to test backwashing behavior at higher pressures.

The large difference between filtration and backwashing flow rate highlights how essential it is to reduce the backwashing duration to a minimum while keeping fouling at a minimum. Based on the experimental observations, no incentive exists to extend the backwashing duration beyond what is required to achieve the desired pressure.

**Figure 8:**
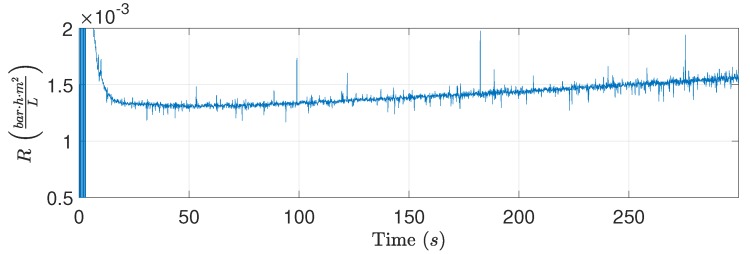
The estimated flow resistance is slowly increasing during backwashing. Experimental conditions: $\Delta P_{b,ref}=-3$ bar.

**Figure 9:**
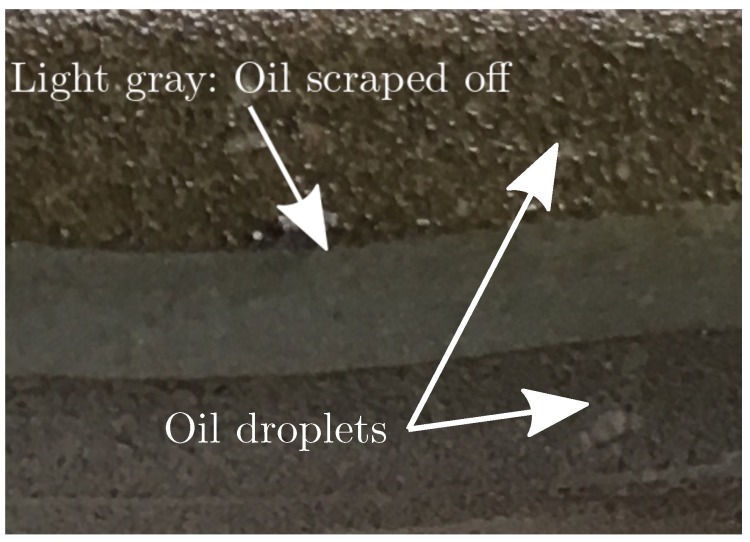
Oil accumulated on the permeate side of the membrane, the light gray color across the membrane is an area where the oil is scraped off.

To ensure the highest degree of removal and minimizing permeate spent backwashing, backwashing is terminated once

(6) $$\Delta P_{b}\left(t\right)\geq \Delta P_{b,ref}$$

is satisfied. Furthermore, extending the backwashing duration beyond 70 s seems to decrease the flux recovered, but additional data are required to be conclusive. The observation could be correlated to the occurrence of fouling during backwashing, as the backwashing media (permeate) contains oil to a degree where fouling on the permeate side of the membrane occurs. Fouling during backwashing is observed in Figure 8, where the estimated resistance increases during backwashing. In addition, a membrane was inspected, and oil fouling was found on the permeate side of the membrane—see Figure 9.

### 3.3. Scheduling Strategy Modifications

Because backwashing termination depends purely on the required time to reach the desired backwashing pressure, the maximization problem defined in Equation (1) is reduced to only be dependent on $t_{f|n}$. As $t_{f|n}$ depends on the backwashing phase and $t_{b|n}$ is independent from the filtration phase, the problem complexity can be reduced by interchanging the filtration and backwashing phases. The suggested optimization problem in Equation (2) is reformulated in terms of backwashing volume ($V_{b|n}$) and the order of the filtration and backwashing phase is switched:

(7) $$j_{avg|n}\left(t_{f|n}\right)=\frac{\int_{t_{b|n}}^{t_{f|n}+t_{b|n}}j_{f|n}\left(t\right)\;dt-V_{b|n}}{t_{f|n}+t_{b|n}},$$

where $t_{b|n}$ and $V_{b|n}$ should remain relatively constant as they largely depend on pump dynamics. Furthermore, $V_{b|n}$ can be calculated after the backwashing phase, and the filtration flux can be integrated online to provide an online estimation of the average flux.

**Figure 10:**
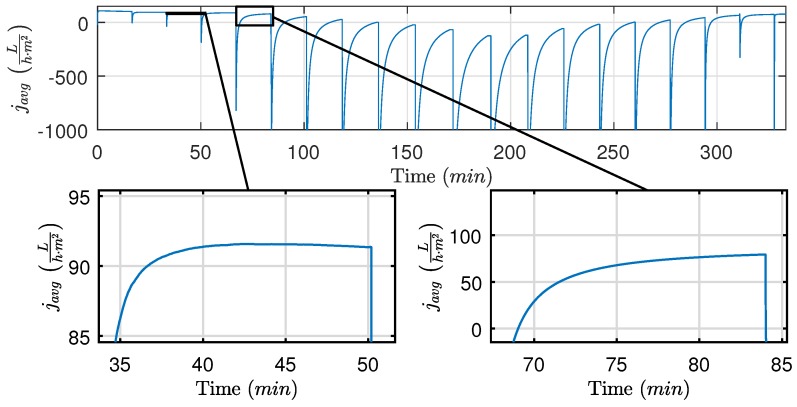
Estimated average flux ($j_{avg}$), based on the data from Figure 5. The bottom left and right figure are the average flux with a backwashing duration of 3.1 s and 9.1 s, respectively.

Based on data from Figure 5, the average flux is calculated and illustrated in Figure 10, where two cases with different backwashing durations are highlighted. For the 3.1 s case (Figure 10 bottom left), the average flux for the filtration cycle peaks at the 42 min mark, whereas the 9.1 s case (Figure 10 bottom right) does not reach maximum flux during the filtration cycle, indicating that the filtration time should be prolonged. Even though the 3.1 s case has the highest maximum average flux for a single filtration cycle, nearly no fouling is removed during the backwashing period, which is confirmed by Figure 5 and Figure 6. To ensure long-time sustained flux and avoid frequent chemical cleaning, it is critical that backwashing pressure is established and the removable fouling is removed before termination of the backwashing phase as written in Equation (6).

To detect if the maximum average flux is reached, the derivative is used and the filtration phase is continued while

(8) $$\frac{d}{dt}j_{avg|n}\left(t\right)\geq 0$$

is satisfied.

### 3.4. Implementation and Discretization

The pilot plant operates at a sample frequency of 100 Hz, but for this implementation the signals are downsampled to 10 Hz to reduce computational and data storage requirements. Discrete time variables, $l_{n}$ and $k_{n}$, are defined to simplify notation for the discretization:

(9a) $$t_{b|n}=l_{n}\cdot T_{s}\;l_{n}\in N,$$

(9b) $$t_{b|n}+t_{f|n}=k_{n}\cdot T_{s}\;k_{n}\in N,$$

where $T_{s}$ is the sample time. The first step in the proposed backwashing scheduling method is backwashing, where the goal is to continue backwashing until the desired pressure is achieved. To reduce noise and potential incorrect switching, a lowpass filter with the specifications from Table 1 is deployed.

As defined in Equation (6), backwashing is terminated once the backwashing pressure is above or equal to the reference. However, as the measurements are noisy and the controller is unable to keep the system at the exact reference, a soft threshold of 95% is combined with a 3 s delay to ensure that backwashing is not unnecessarily prolonged or triggered by noise. As a result, backwashing is continued while:

(10) $$\begin{array}{cc} (\Delta P_{b}\left(l_{n}T_{s}\right)0.95<\Delta P_{b,ref}) & \;\wedge \\ (\Delta P_{b}\left(\left(l_{n}-30\right)T_{s}\right)0.95<\Delta P_{b,ref}) & \end{array}$$

is satisfied, where $\Delta P_{b,ref}$ is the reference for the backwashing pressure controller, which is kept constant. While the backwashing is executed, the volume of backwashing media spent is calculated online as:

(11) $$V_{b|n}=\sum_{i=0}^{l_{n}}j_{b|n}\left(iT_{s}\right)T_{s}.$$

The second step is to continue filtration while the average flux is increasing. For that purpose, Equation (7) is discretized to:

(12) $$j_{avg|n}\left(k_{n}\right)=\frac{\sum_{i=l_{n}}^{k_{n}}j_{f|n}\left(iT_{s}\right)T_{s}-V_{b|n}}{k_{n}T_{s}}.$$

The termination of the filtration phase relies on Equation (8), which can be approximated as:

(13) $$\frac{d}{dt}j_{avg|n}\left(t\right)\approx j_{avg|n}\left(hT_{s}\right)-j_{avg|n}\left(\left(h-1\right)T_{s}\right)>0,$$

where *h* is the current sample. The approximation can be formulated as:

(14) $$\begin{array}{cc} \frac{d}{dt}j_{avg|n}\left(t\right) & \approx \frac{\sum_{i=l_{n}}^{k_{n}-1}j_{f|n}\left(iT_{s}\right)T_{s}+j_{f|n}\left(k_{n}T_{s}\right)T_{s}-V_{b|n}}{k_{n}T_{s}}\\ & -\frac{\sum_{i=l_{n}}^{k_{n}-1}j_{f|n}\left(iT_{s}\right)T_{s}-V_{b|n}}{\left(k_{n}-1\right)T_{s}}>0. \end{array}$$

The inequation can then be simplified to:

(15) $$j_{f|n}\left(k_{n}T_{s}\right)-\frac{\sum_{i=l_{n}}^{k_{n}-1}j_{f|n}\left(iT_{s}\right)T_{s}+V_{b|n}}{\left(k_{n}-1\right)T_{s}}>0.$$

The condition defined in Equation (8) can be written as:

(16) $$j_{f|n}\left(k_{n}T_{s}\right)>\frac{\sum_{i=l_{n}}^{k_{n}-1}j_{f|n}\left(iT_{s}\right)T_{s}+V_{b|n}}{\left(k_{n}-1\right)T_{s}}.$$

Based on Equations (10) and (16), the proposed backwashing scheduling algorithm is formulated as in Algorithm 1.
**Algorithm 1** Proposed scheduling algorithm.
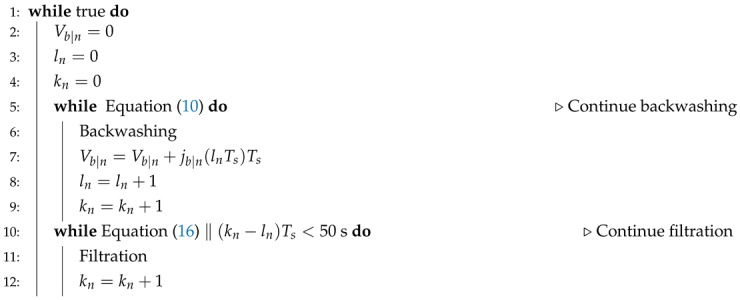


The suggested backwashing scheduling algorithm maximizes the net flux production over a filtration cycle and completely avoids any model estimation process, ensuring easy implementation. $t_{b|n}$ is determined by the required time to reach the desired backwashing pressure plus 3 s, avoiding being triggered by noise and ensuring removed oil exits the system before the filtration phase is reinitiated. Simultaneously, the volume of permeate used for backwashing this cycle is calculated based on flow measurements, which is used in Line 10 Equation (16). After the backwashing phase, the filtration phase is continued while the calculated average flux increases.

The algorithm relies on the derivative of $j_{avg|n}$, which is sensitive to noise, thus the measurements are filtered using a lowpass filter to reduce high frequency noise. The lowpass filter introduces a delay, depending on filter type and order, potentially delaying the switch between backwashing and filtration and thereby reducing performance.

**Figure 11:**
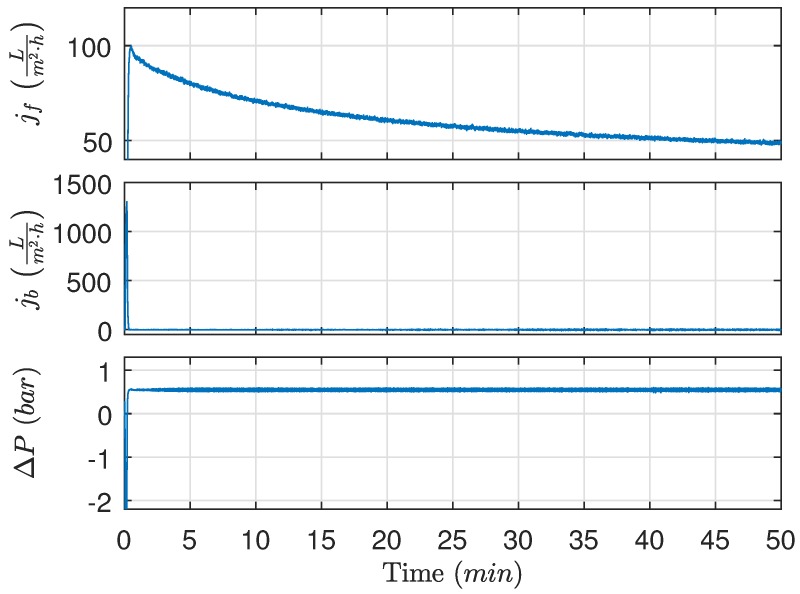
Single filtration cycle, initiated with backwashing and followed by filtration. Experimental conditions: $t_{f}=3000$ s, $t_{b}=10$ s, $Q_{cf,ref}=1\;\frac{m}{s}$, $\Delta P_{f,ref}=0.55$ bar, and $\Delta P_{b,ref}=-2$ bar.

The algorithm is implemented in Simulink and an experiment is conducted to evaluate the performance of the online numerically calculated $j_{avg|n}$ and $\frac{d}{dt}j_{avg|n}$—see Figure 11. The experiment is divided into two phases. Firstly, backwashing is carried out according to the algorithm. Secondly, the filtration phase is carried out beyond what would initiate backwashing, such that the performance of the online numerically calculated $j_{avg|n}$ and $\frac{d}{dt}j_{avg|n}$ could be evaluated and observed in the transition phases. Based on the experiment, two problems are observed.

**Figure 12:**
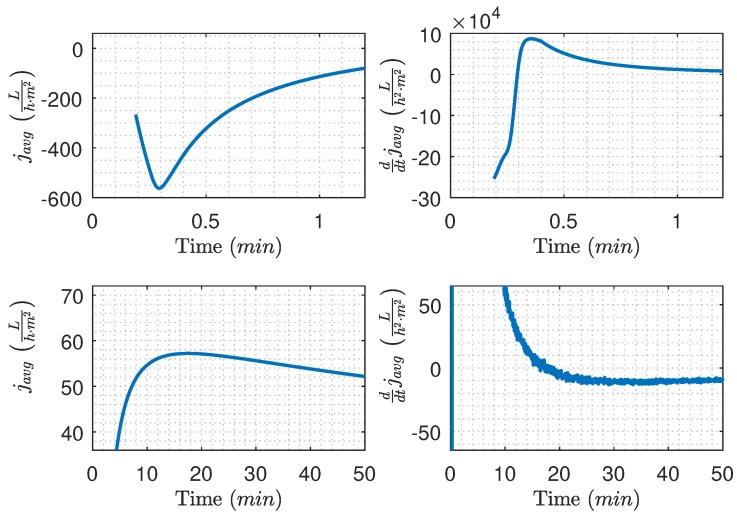
Estimated $j_{avg}$ and its derivative, where the top figures show the effect of filter initialization and the transition between backwashing and filtration on $j_{avg}$ and $\frac{d}{dt}j_{avg}$. The bottom figures show the values of $j_{avg}$ and $\frac{d}{dt}j_{avg}$ for the entire experiment—based on data from Figure 11.

Firstly, initialization of the filtration system and lowpass filter, combined with switching between backwashing and filtration, causes the algorithm to initiate backwashing prematurely as shown in Figure 12 (the two top figures). The problem can be avoided by enforcing a minimum filtration time of 50 s, as described in Algorithm 1. Secondly, the derivative amplifies the measurement noise, as highlighted in Figure 12 (bottom right). The amplified noise could initiate backwashing before the true $\frac{d}{dt}j_{avg}$ reaches zero.

**Figure 13:**
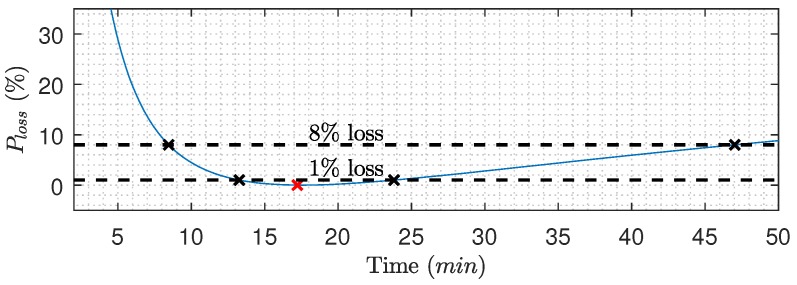
Average flux loss as a function of time (Equation (17)), where the red cross is the optimal point ($0\%\;P_{loss}$)—based on data from Figure 11.

A premature termination of the filtration phase will cause a loss in overall production, but to which degree is unclear. To quantify the consequence of an untimely termination of the filtration phase, the production loss, as defined in Equation (17), is plotted as a function of filtration time in Figure 13:

(17) $$P_{loss}\left(t_{n}\right)=\frac{max\left(j_{avg|n}\left(t\right)\right)-j_{avg|n}\left(t_{n}\right)}{max(j_{avg|n}\left(t\right))}.$$

Figure 13 highlights the filtration time ranges with a maximum of 1% and 8% production loss compared to the optimal point (red cross), respectively. The loss in permeate production is not symmetric around the optimal point and favors late over early termination. Furthermore, Table 2 shows the filtration time ranges at which the filtration phase must be terminated if $P_{loss}$ should be less than the given percentages. For example, filtration must be terminated within a 3 min window if $P_{loss}$ should be less than 0.1%. The table with respect to filtration duration and production loss highlights the irrelevance of the 4.25 s filter delay on the flow measurements. The estimated production losses and time windows are case dependent, and the exact values will vary with membrane conditions, feed properties, and process conditions.

**Figure 14:**
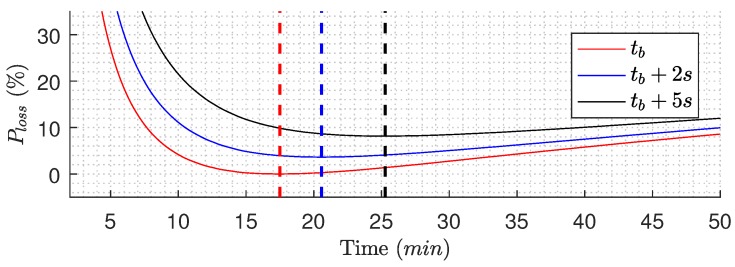
Estimated average flux loss as a function of time (Equation (17)) for different backwashing duration extensions, where the $max(j_{avg|n}\left(t\right))$ term is the maximum $j_{avg}$ across time and different backwashing durations. The dashed lines are the optimal $t_{f}$ for the different backwashing durations—based on data from Figure 11.

Based on the experiment shown in Figure 11, the effect of extending the backwashing duration is estimated. To estimate the potential loss caused by extending the backwashing duration, it is assumed that the extended duration provides no additional fouling removal, and the backwashing flow rate can be maintained for the extended duration. As the optimal filtration time changes as the backwashing time is extended, the average flux loss as a function of time for different extensions is shown in Figure 14. By extending the backwashing duration by 5 s, the estimated loss using the proposed algorithm is 8.8%. However, the loss caused purely by extending the backwashing duration is reduced as the filtration duration is extended, and, if the filtration is allowed to continue for the full duration of the experiment (50 min), the loss is reduced to 3.5%.

### 3.5. Scheduling Results

**Figure 15:**
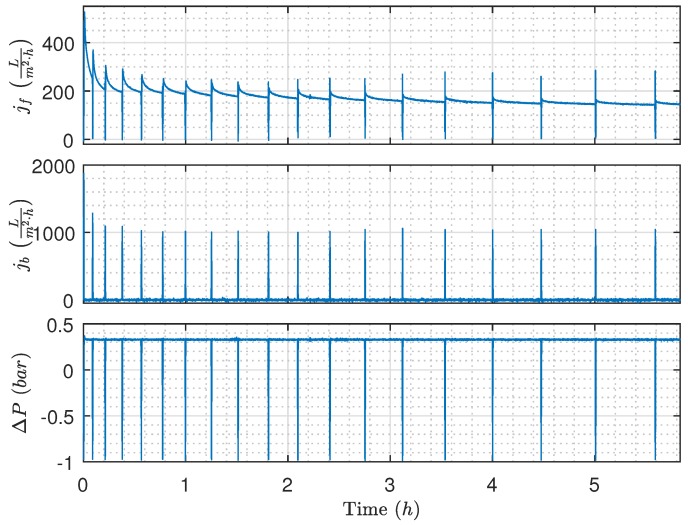
Filtration flux, backwashing flux, and TMP while deploying the proposed algorithm. Experimental conditions: $Q_{cf,ref}=1\;\frac{m}{s}$, $\Delta P_{f,ref}=0.33$ bar, and $\Delta P_{b,ref}=-1$ bar.

**Figure 16:**
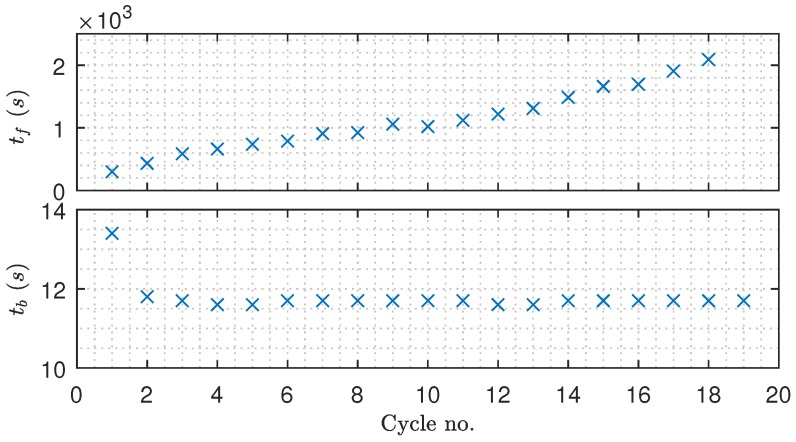
The filtration and backwashing durations from the experimental validation of the proposed algorithm—based on data from Figure 15.

**Figure 17:**
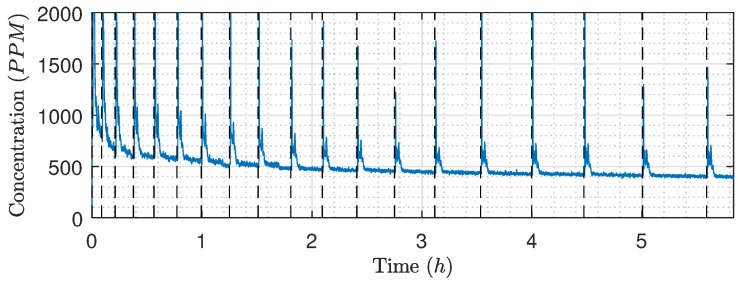
Inlet concentration during the validation experiment (Figure 15 and Figure 16), according to the TD-4100XD. The black dashed lines indicate filtration termination and backwashing initialization.

The proposed algorithm is implemented on the pilot plant, where new membranes are installed to highlight how the algorithm adapts the durations as irreversible fouling accumulates. The results are presented in Figure 15, Figure 16 and Figure 17. During the validation experiment, the CFV, TMP, and the backwashing pressure are maintained at levels indicated in Figure 15 by deploying feedback control, and the temperature is 21 °C. To ensure the proposed algorithm is able to adapt to changes, the OiW concentration (likely also droplet size), irreversible fouling, and the feed flow rate varied during the experiment.

The filtration and backwashing durations throughout the experiment are shown in Figure 16, where the backwashing duration remains relatively constant, and the filtration duration is slowly and steadily increasing as irreversible fouling accumulates in the newly installed membranes. The fact that the backwashing frequency is decreased as irreversible fouling occurs contradicts the results from the TMP/flux threshold-based method described in [12], where the backwashing frequency is increased as irreversible fouling accumulates. It is conceivable that accounting for irreversible fouling growth in the backwashing fouling model would increase the backwashing frequency to avoid accumulation of irreversible fouling. Furthermore, the constant TMP control mode can also contribute to the decreasing backwashing frequency. However, as irreversible fouling accumulates, the flux is reduced as a consequence of constant TMP control. As the flux is reduced so does the fouling growth rate and the need for backwashing the membrane. Consequently, the backwashing frequency is reduced. Despite the fouling state of the membranes, the cost of backwashing is the same and, as the filtration flux is reduced, filtration must be carried out for a longer period in order to justify the permeate cost of backwashing.

The feed concentration in Figure 17 showed the same decreasing tendency as the permeate flow rate, indicating that the decrease in concentration could be caused by oil accumulating in the membranes. Furthermore, the concentration peaks occurring after each backwash are unrelated to the storing of the permeate after each backwash as the concentration should be decreasing and not increasing. The concentration peaks are likely a combination of two factors: firstly, the sensor is drained for water, which allows air inside the view cell during backwashing. Secondly, it is observed that OiW concentrations this small are difficult to measure and the instrument is sensitive to the dynamic transition between backwashing and filtration. The decreasing OiW concentration during the experiment does probably affect the droplet size distribution of the feed. Ideally, the distribution should be measured over the course of the validation experiment. However, the ViPA is based on video microscopy and the size of the view cell means that only a very small fraction of the flow can be observed. Consequently, the sensor is required to run for several hours under steady-state conditions to gather enough samples to create a representative size distribution, and, as the conditions are constantly changing during the experiment, the relationship between OiW concentration and size distribution is unknown.

The mean outlet concentration for the entire experiment was constantly −9.7 ppm, according to the TD-4100XD. The observed negative concentration is caused by a calibration offset, which confirms that the absolute precision is not to be completely trusted, but it does provide an indication of how the feed concentration changes over the course of the experiment.

## 4. Conclusions

The fouling removal during backwashing was concluded to be unobservable, making online estimation of the resistance difficult with the available measurements. While no reduction in resistance was observed during backwashing, a reduction resistance could be observed once the filtration phase was initiated. Furthermore, prolonging the backwashing duration beyond what is required to achieve the desired TMP resulted in no additional flux recovery, as such permeate used by backwashing could be reduced by optimizing the pressure delivering capabilities of the backwashing supply system.

The backwashing flux could be up to eight times higher than the filtration flux, emphasizing the criticality of the backwashing duration. To quantify, a 5 s backwashing extension causes a production loss of between 3.5% to 8.8%, whereas an extension of 13.4 min for filtration duration only causes a production loss of 3%. Evidently, the backwashing duration is more time critical. Consequently, the delay introduced can impact permeate production, and efforts should be aimed towards reducing any switching delay. In particular, the filter delay combined with the 3 s delay defined in Equation (10) can decrease the overall performance. It is conceivable that the lowpass filter would be sufficient to avoid backwashing termination caused by measurement noise.

Based on the observed fouling behavior, a scheduling algorithm is proposed and tested on the pilot plant. The algorithm is limited to filtration systems with measurements and subject to constant TMP control, and modification is required to adapt the algorithm to non-constant TMP control. A benefit of the suggested algorithm is that both the backwashing and filtration durations are completely dependent on measurements, requiring no model and allowing for fast adaptation to changes in irreversible fouling and OiW concentration. Longer or more frequent backwashing could conceivably reduce irreversible fouling growth, as the correlation between backwashing scheduling and irreversible fouling is not considered. Currently, only the current cycle is considered for optimization. However, to account for long-term operation, backwashing is continued until the backwashing pressure is established and all removal fouling is removed.

In future work, the suggested algorithm should include irreversible fouling and backwashing pressure into the fouling removal behavior, to reduce irreversible fouling and ensure that the maximum degree of fouling is removed. As the suggested algorithm completely relies on measurements, sensor faults or calibration error are critical and Kalman filter based methods could be deployed to detect sensor failure [32]. Lastly, the OiW mixture used in the study is a simplification of the real offshore PW neglecting the chemical and biological interaction present in real PW, potentially changing the fouling behavior. To ensure that the method is deployable for PWT, the difference must be compared. The validation is carried out at different concentrations and irreversible fouling levels, but future work should also include different oil compositions.

## Figures and Tables

**Table 1:** Finite impulse response (FIR) Lowpass filter specifications.

Type	FIR
Passband	0.1 Hz
Stopband	0.5 Hz
Minimum stopband attenuation	80 dB
Group delay	42.5 samples
Sample rate	10 Hz

**Table 2:** Maximum production loss at time intervals, where time interval is defined as: *Optimal time* + [*time range with the corresponding $P_{loss}$*].

Maximum Allowed $P_{\mathrm{loss}}$ (%)	Time Intervals (min)
0	17.2
0.1	17.2+[−1.1,+2.2]
1	17.2+[−3.9,+6.6]
2	17.2+[−5.3,+10.2]
3	17.2+[−6.2,+13.4]
4	17.2+[−6.9,+16.6]
6	17.2+[−8,+23]
8	17.2+[−8.7,+29.8]
